# A 10-meter annual time-series of plantation and natural forests in Southeast Asia (2017–2024)

**DOI:** 10.1038/s41597-026-07450-6

**Published:** 2026-05-20

**Authors:** Cheng Rui, Chiwei Xiao, Yunfeng Hu

**Affiliations:** 1 Institute of Geographic Sciences and Natural Resources Research, Chinese Academy of Sciences, Beijing, China; 2 College of Resources and Environment, University of Chinese Academy of Sciences, Beijing, China; 3 Key Laboratory of China-ASEAN Satellite Remote Sensing Applications, Ministry of Natural Resources of the People’s Republic of China, Nanning, China; 4 Jiangsu Center for Collaborative Innovation in Geographical Information Resource Development and Application, Nanjing, China

## Abstract

Developing high-precision spatial datasets of plantation forests (PF) and natural forests (NF) in Southeast Asia (SEA) is critical for understanding tropical forest loss, biodiversity degradation, agroforestry management, and regional sustainable development. However, existing land cover products and related forest thematic data often fail to distinguish between PF and NF, and often lack the high spatial resolution and consistent temporal frequency. Here, we generated an annual 10-m resolution PF and NF distribution dataset for SEA (or SEA-PNF) from 2017 to 2024, using an iterative sample-refinement strategy integrating multi-source data and temporal features. By integrating Google Alpha Earth imagery, Random Forest classifiers, and a Hidden Markov Model (HMM)-based temporal optimization algorithm, we effectively addressed classification challenges inherent to complex tropical landscapes. Validation results of our SEA-PNF demonstrate that both the producer’s accuracy and the user’s accuracy for PF and NF exceed 0.95. This dataset reveals the fine-scale spatio-temporal characteristics of PF and NF in SEA, providing essential data support for regional agroforestry changes and ecological assessment.

## Background & Summary

Amidst global efforts to combat climate change and biodiversity loss, accurate monitoring and quantification of spatio-temporal forest dynamics are essential for achieving carbon neutrality and sustainable development goals^1–4^. As key regulators of the global carbon cycle and biodiversity, tropical forests have garnered unprecedented attention. Southeast Asia (SEA) hosts nearly 15% of global tropical forests^5^ and suffers from one of the highest deforestation rates globally^6,7^. Unlike South America, where ranching is the primary driver, forest loss in SEA is driven primarily by global commodity demand and agricultural expansion^6,8^. This process is characterized by the systematic clearance of biodiversity-rich, high-carbon natural forests (NF) and their conversion into intensively managed plantation forests (PF), primarily consisting of oil palm, rubber, banana, and eucalyptus^7,9,10^. Fundamental differences exist between NF and PF regarding ecological function, carbon storage, and biodiversity carrying capacity^11^. Although several land cover products include a general “forest” class^12,13^, they currently fail to differentiate between PF and NF. Consequently, it remains challenging to assess carbon neutrality or biodiversity loss accurately, or to formulate effective conservation policies, due to the varying proportions of these forest types^14,15^.

Despite the urgent demand to distinguish NF and PF, current datasets are constrained by coarse spatial resolution and insufficient temporal frequency. Early global and/or regional forest mapping efforts largely depended on coarse-resolution data (e.g., 500-meter MODIS)^16^. Although suitable for capturing continental-scale trends, such data fail to resolve the highly fragmented and heterogeneous landscapes across SEA. Recent global PF products have improved resolution to 250-meter^3^, yet this scale remains insufficient for pixel-level or plot-level management. With the opening of the Landsat archive, 30-m products have become mainstream, such as the JRC Global Forest Type 2020 map (GFT2020)^17^, the NPF30-2020 dataset^18^, and the 1990–2020 China PF/NF dataset^19^. However, most of these products either provide static snapshots without annual dynamics or remain limited to 30-m resolution, thereby failing to capture fine-scale PF expansion. Furthermore, the widely used Spatial Database of Planted Trees (SDPT), being a compilation database^20^, suffers from inconsistencies in data sources, years, and definitions across different countries. Existing research confirms that in the agriculturally fragmented landscapes of SEA, current datasets show poor spatial consistency and high uncertainty in distinguishing NF from PF^16,21^. Recently, 10-m data from Sentinel satellites have enabled finer monitoring^22^. However, existing global 10-m products, such as ESA WorldCover^12^ and ESRI Land Cover (2017–2024)^23^, continue to aggregate NF and PF into a single mixed “forest” class.

In all, there is a lack of publicly available datasets that provide high spatio-temporal resolution while effectively distinguishing NF from PF. Here, the latest 2017–2024 Google Alpha Earth dataset^24^ was used to construct a 10-m annual thematic dataset for SEA. This dataset of 10-m resolution PF and NF (or SEA-PNF) facilitates the fine-scale analysis of the interplay between PF expansion and NF retreat, providing crucial data support for regional ecological assessments and forest resource management.

## Methods

### Study area

**Fig. 1:**
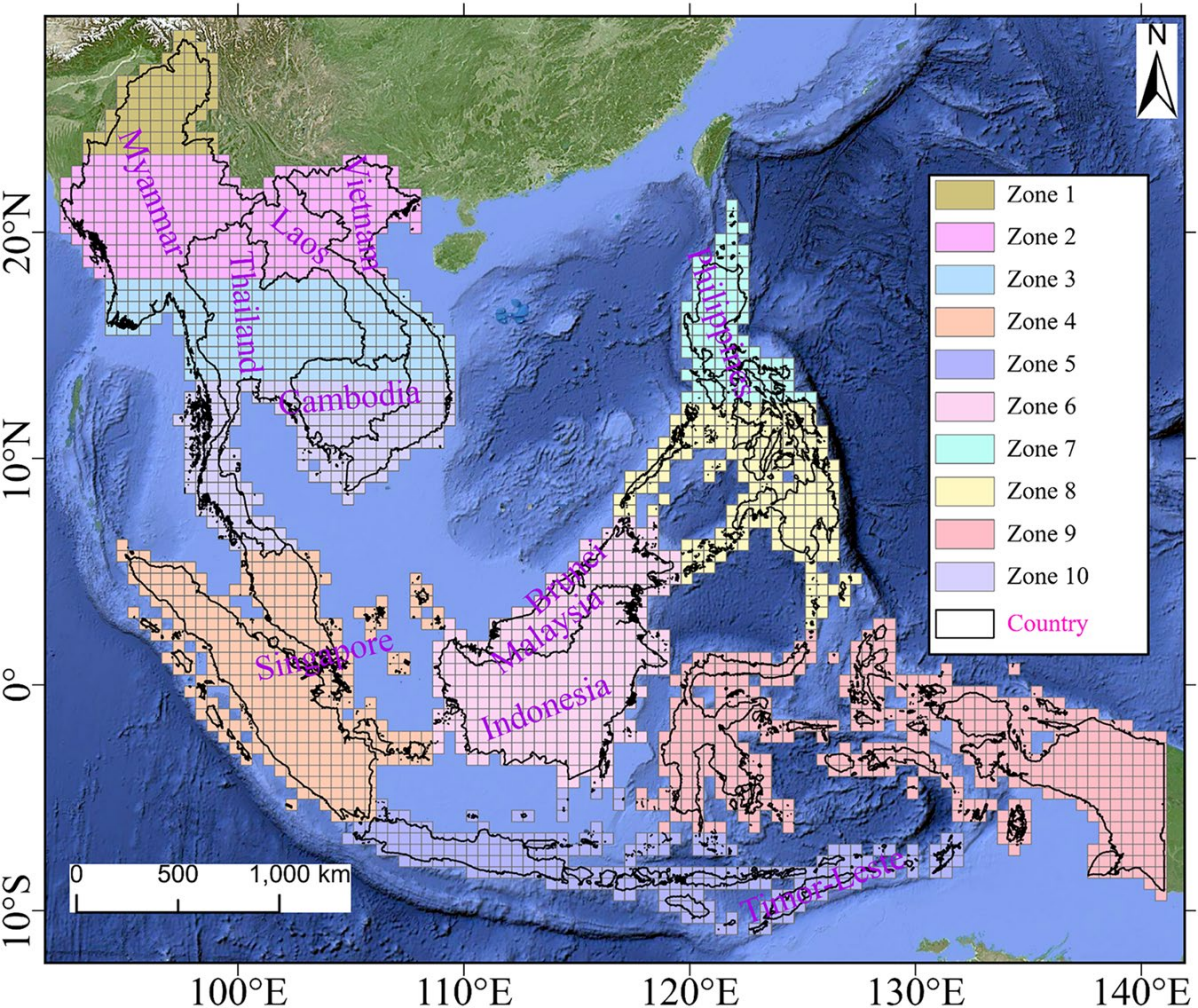
Study area map of SEA, illustrating the 10 latitude-based geographical classification zones (colored grids) and national boundaries.

This study covered SEA, comprising 11 countries, namely Myanmar, Thailand, Laos, Vietnam, Cambodia, Malaysia, Indonesia, the Philippines, Brunei, Timor-Leste, and Singapore. The region featured complex terrain and diverse climates, hosted rich tropical rainforest resources, while also facing drastic land-use changes^25^ (Fig. 1).

### Definitions of forest types

From an Earth observation perspective, PF (e.g., oil palm, rubber, eucalyptus) were defined as intensively managed tree crops characterized by highly uniform spectral signatures and synchronized temporal phenology. In contrast, NF were defined as naturally regenerated ecosystems exhibiting complex canopies with heterogeneous spectral textures and stable temporal records^18^.

### Input Data

Google Alpha Earth: The Google Alpha Earth satellite image mosaic (2017–2024) was provided by Google and served as the primary data source for classification, providing cloud-free annual imagery with 64 bands at 10-m resolution. Unlike traditional spectral bands, it provided 10-m cloud-free annual imagery where each pixel was a 64-dimensional embedding vector generated by the Alpha Earth Foundations model (v2.1), encoding multi-source temporal trajectories^24^. It was accessed via the Google Earth Engine (GEE) platform (ImageCollection ID: GOOGLE/SATELLITE_EMBEDDING/V1/ANNUAL). Spatial Database of Planted Trees (SDPT v2.0): Published by the World Resources Institute (WRI), this dataset aggregates plantation vector data from various countries and was used as the primary source for PF samples. SDPT was accessed via 10.46830/writn.23.00073. NPF30-2020: Published by Fudan University, this global 30-m product was obtained via 10.5281/zenodo.10701417. And NPF30-2020 was the first global 30-m PF and NF classification product, with an overall global accuracy of approximately 85%. Global CCDC V1: Google-developed global change parameters generated using the Continuous Change Detection and Classification (CCDC) algorithm. This data was used to exclude sample points that underwent drastic changes and was accessed via GEE (ImageCollection ID: projects/gee-ccdc/assets/global_ccdc_v1). ESRI Land Cover (2017–2024): This dataset was provided by ESRI and accessed via ESRI Living Atlas (https://livingatlas.arcgis.com/landcoverexplorer/) and utilized for the preliminary screening of stable sample points.

### Sample Refinement

**Fig. 2:**
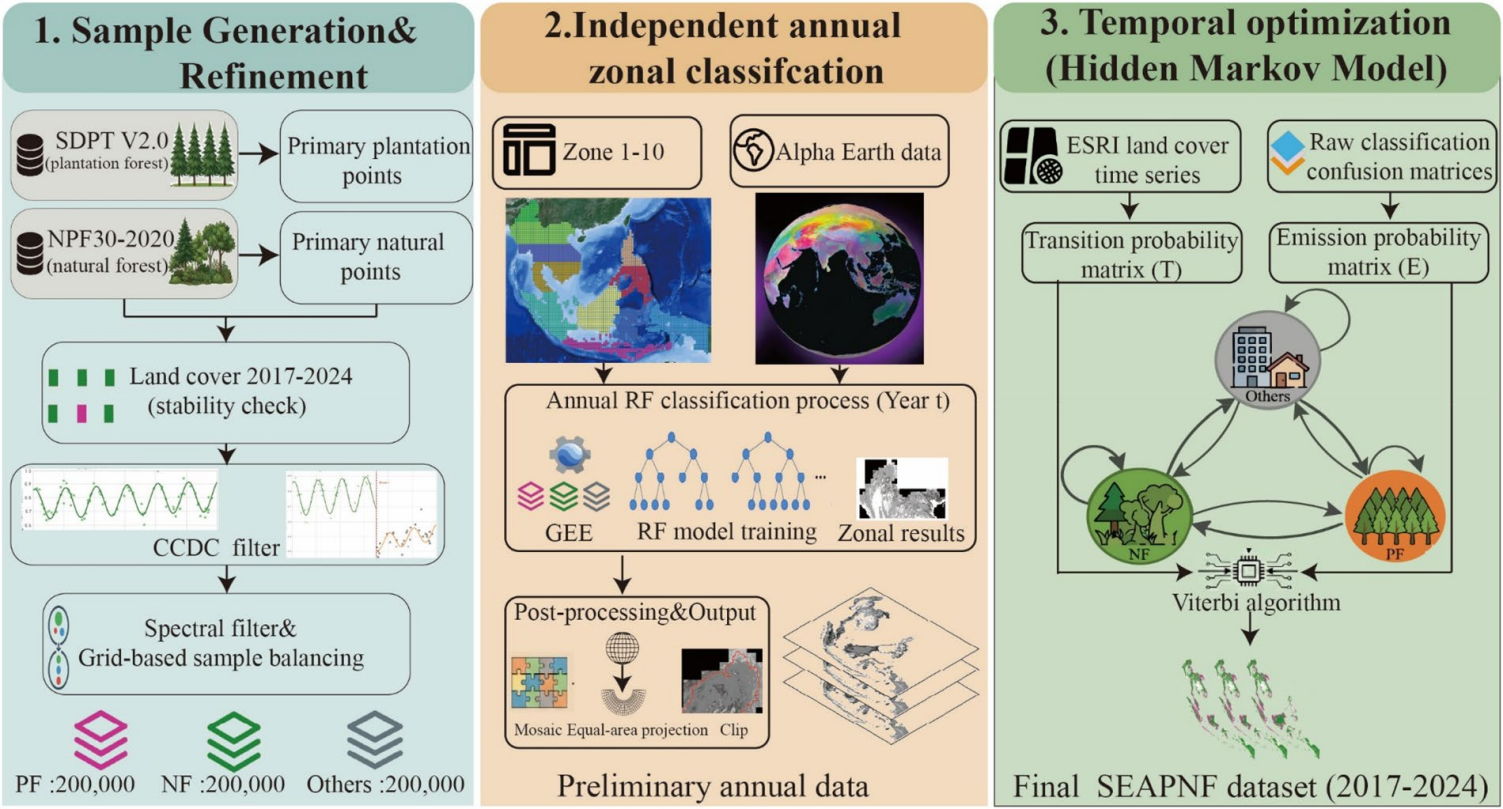
The workflow of the plantation and natural forests (PF & NF) classification in SEA.

To address inherent label noise and differing classification definitions across source products, and to align the samples with our unified earth observation definitions^18,26–28^, we designed a multi-stage automated iterative sample-generation strategy to strictly filter the training sets both spatiotemporally and spectrally (Fig. 2).

1. Initial Sample Generation: For PF, in countries covered by SDPT, random points were generated at a density of 1 point per km^2^ within SDPT v2.0 vector boundaries, masked by ESRI Land Cover (forest) and NPF30-2020 (PF class). In countries without SDPT coverage, points were randomly sampled from the NPF30-2020 PF class at a rate of 1 point per 100 pixels. NF samples were drawn from the ESRI Land Cover “Trees” class (excluding SDPT and NPF30-2020 PF areas) at 1 point per 1000 pixels. Non-forest samples were drawn from ESRI non-tree classes at 1 point per 50 pixels.
2. Temporal Stability Screening: We retained points that remained stable in the ESRI Land Cover classification from 2017 to 2024. Using Google Global CCDC V1, we specifically extracted the timing of spectral breaks (tBreak) and the total number of breaks (nBreaks) to evaluate the temporal stability of each sample point. Then we excluded points with multiple abrupt changes between 2000 and 2019. Specifically, PF samples were retained only if the abrupt change occurred after 2010; NF samples were retained if they had fewer than two changes, with the last change occurring before 2010.
3. Spectral Cleaning: We calculated the spectral features of each sample point using all 64 semantic embedding dimensions generated by the corresponding annual Google Alpha Earth model. A stringent statistical test based on the Mahalanobis Distance^29^ and the Chi-square distribution was used to remove extreme outliers (P < 0.001). Additionally, 70% of high-deviation samples (0.001 < P < 0.01) were randomly pruned^30^.
4. Sample Balancing: To generate year-specific sample sets and address spatial autocorrelation, spatial and quantitative balancing was performed at a 0.5° × 0.5° grid scale for each year to align the ratio^31^ of PF, NF, and Non-forest samples close to 1:1:1. This grid-based stratification ensureed samples were evenly distributed across the landscape rather than spatially clustered, effectively mitigating spatial autocorrelation. This resulted in a high-quality training set containing approximately 600,000 samples.

### Zonal independent annual classification

We employed a Random Forest (RF) classifier^32^ implemented entirely on the Google Earth Engine (GEE) platform for annual classification using all 64 bands of the Google Alpha Earth imagery as the feature space. The RF model was configured with 300 decision trees to balance computational efficiency and large-scale classification accuracy, and was trained using an 80% and 20% random split of the samples for training and independent testing, respectively. To accommodate the heterogeneity of forests across SEA, we divided the study area into 10 geographical zones^33^ and trained independent annual RF models for each zone. Finally, predictions were generated for each year from 2017 to 2024 to produce preliminary annual classification maps.

### Post-processing by hidden markov model

Independent annual classifications can produce logical temporal errors, such as spurious “NF-PF-NF” transitions. To mitigate this, we innovatively introduced a Hidden Markov Model (HMM) for temporal smoothing^34,35^. The model comprised three core matrices: (1) Transition Probability Matrix (T): We derived T by calculating general “Forest” and “Others” transition frequencies from the 2017–2024 ESRI dataset, and then proportionally partitioning them into specific PF and NF probabilities based on our initial Random Forest classifications. The matrix (rows/cols: PF, NF, Others) is defined as:

$$T=\left[\begin{array}{ccc}0.94 & 1\times {10}^{-9} & 0.06\\ 0.02 & 0.96 & 0.02\\ 0.045 & 0.01 & 0.945\end{array}\right]$$

We set the probability of PF converting to NF (${P}_{{PF}\to {NF}}$) to a negligible value ($1\times {10}^{-9}$) to reflect ecological reality: plantations typically require harvesting (transition to non-forest) or long-term succession to recover into NF, a process that could not occur within a single year^36^. (2) Emission Probability Matrix (E): Derived from the confusion matrix of the initial RF classification results^34^, representing the conditional probability of observing a class given the true state:

$$E=\left[\begin{array}{ccc}0.9132 & 0.0853 & 0.0014\\ 0.0973 & 0.8999 & 0.0029\\ 0.0070 & 0.0042 & 0.9888\end{array}\right]$$

(3) Initial Probabilities (π): These were set based on the initial area percentages of PF, NF, and Others in the study area:$\,\pi =\left[0.1267,0.3848,0.4885\right]$. Finally, the Viterbi algorithm^37^ was applied to identify the globally optimal sequence of hidden states, correcting unreasonable short-term fluctuations and generating a spatiotemporally consistent SEA-PNF dataset for 2017–2024.

## Data Records

The dataset^38^ generated in this study has been deposited in the figshare repository and contains annual classification maps of PF and NF in SEA from 2017 to 2024. Data are stored in GeoTIFF format under the WGS84 coordinate system (EPSG:4326), with a spatial resolution of 10-m (reprojected to an equal-area projection for area calculations). Pixel values are defined as: 1 (i.e., PF), 2 (i.e., NF), 3 (i.e., non-forest), and 0 (i.e., no data).

### Data overview

The dataset comprises eight annual NF and PF maps (2017–2024) covering the entire SEA region at 10-m resolution (Fig. 3), and the classification results spatially depict the distribution of PF and NF. Figure 3i further presents the area confidence intervals for PF and NF, calculated using the method proposed by Olofsson *et al*.^39^.

**Fig. 3:**
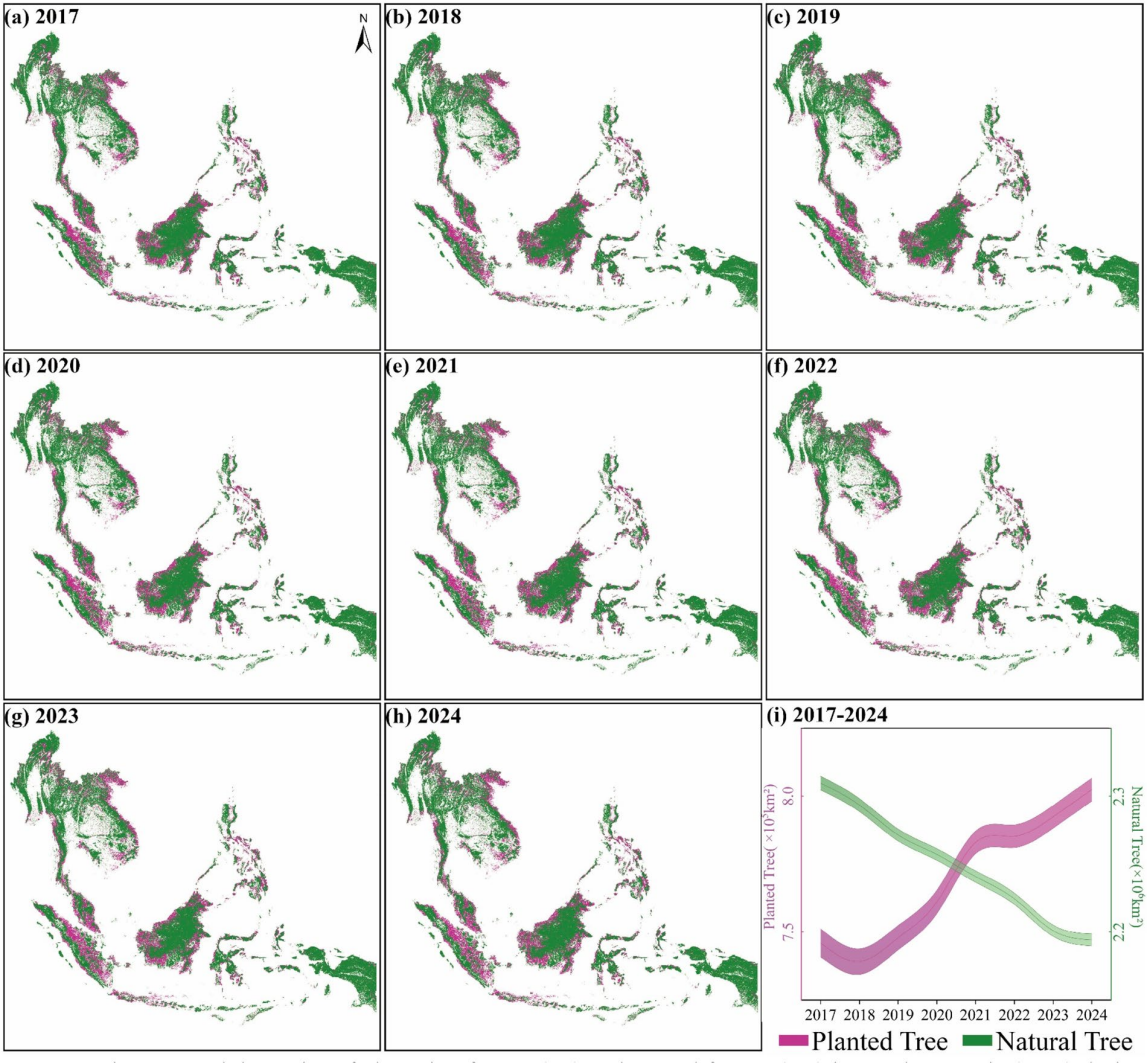
Spatio-temporal dynamics of plantation forests (PF) and natural forests (NF) in Southeast Asia (SEA) during 2017–2024. (**a**–**h**) Annual spatial distribution of PF and NF from 2017 to 2024 in SEA. (**i**) Annual area changes of PF and NF during 2017–2024 in SEA.

## Technical Validation

### Accuracy assessment

**Fig. 4:**
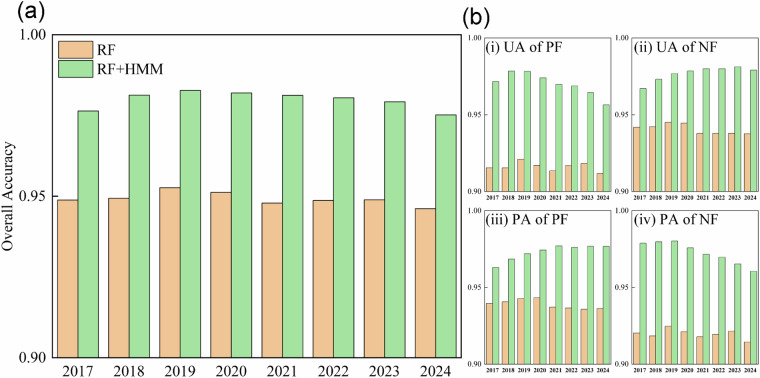
Comparison of classification accuracy between the baseline Random Forest (RF) model and the HMM-optimized model. (**a**) Overall Accuracy. (**b**) Detailed User’s Accuracy (UA) and Producer’s Accuracy (PA) for PF (i and iii) and NF (ii and iv).

To comprehensively assess dataset quality, we adopted a stratified random sampling strategy, retaining approximately 120,000 independent samples (20% of the total) that were excluded from model training^40^. We calculated confusion matrices, Overall Accuracy (OA), User’s Accuracy (UA), and Producer’s Accuracy (PA) for both the initial Random Forest model and the HMM-optimized results. Validation results (Fig. 4) indicate that the initial classification achieved high accuracy, with an average OA of approximately 94.9% for 2017–2024. Non-forest (Class 3) identification was the most robust, with UA and PA remaining stable at around 99%. PF (Class 1) and NF (Class 2) showed slightly lower accuracy (UA: 91–92%, PA: 92–94%), indicating some “salt-and-pepper” noise and class confusion in complex mixed areas. The introduction of HMM post-processing significantly enhanced classification performance, particularly by resolving temporal inconsistencies. As shown in Fig. 4a, HMM optimization improved the OA across all years, averaging ~98.0% (an increase of ~3 percentage points), peaking at 98.3% in 2019. The improvement was most pronounced for PF (Fig. 4b): following HMM processing, the UA for PF jumped from ~91.6% to over 96.5% (reaching 97.8% in 2018), and PA increased from ~93.8% to over 97.0%. This suggests that the HMM effectively filtered false changes caused by phenological fluctuations or cloud contamination. Similarly, NF accuracy improved (Fig. 4b), with UA rising from ~94.0% to ~97.5% and PA from ~92.0% to ~97.0%.

### Inter-comparison with FAO Statistics

**Fig. 5:**
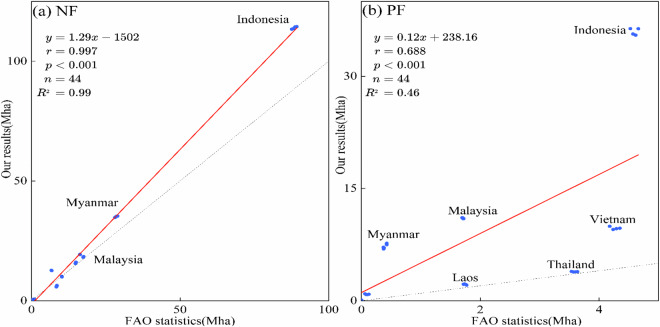
Comparison of aggregated areas for (**a**) natural forests and (**b**) plantation forests derived from this study versus FAO FRA 2020 country reports (${\boldsymbol{x}}$: FAO statistical area; ${\boldsymbol{y}}$: mapped area; ${\boldsymbol{r}}$: Pearson correlation; ${\boldsymbol{p}}$: significance level; ${\boldsymbol{n}}$: sample size; ${{\boldsymbol{R}}}^{{\boldsymbol{2}}}$: coefficient of determination).

To evaluate reliability at the national scale, we aggregated the annual classification results for 2017–2020 (Table 1). We compared them against 88 data pairs from the FAO Global Forest Resources Assessment 2020 (FRA 2020) official statistics^41^. As shown in Fig. 5, the NF class showed extremely high consistency with FAO statistical data (${R}^{2}=0.99$), with most data points clustering closely around the 1:1 line. In contrast, the comparison for PF showed a statistically significant relationship but a lower coefficient of determination (${R}^{2}=0.46$), a slope of only 0.12, and a Pearson correlation coefficient (r) of 0.69.

**Table 1:** Annual area of plantation forests and natural forests by country in SEA from 2017 to 2024 (10^4 ^ km^2^).

Country	Class	2017	2018	2019	2020	2021	2022	2023	2024	Trend
Vietnam	PF	9.79	9.43	9.58	9.67	9.81	9.68	9.75	9.90	
	NF	10.29	10.15	10.01	9.93	9.83	9.76	9.62	9.69	
Myanmar	PF	7.26	7.36	7.51	7.66	7.79	7.89	8.17	8.34	
	NF	35.60	35.40	35.07	34.77	34.47	34.24	34.01	34.12	
Malaysia	PF	10.86	10.92	11.05	11.09	11.26	11.33	11.52	11.51	
	NF	18.49	18.39	18.22	18.10	17.94	17.80	17.62	17.53	
Timor-Leste	PF	0.08	0.09	0.10	0.07	0.08	0.08	0.12	0.13	
	NF	0.73	0.72	0.72	0.70	0.70	0.70	0.70	0.72	
Thailand	PF	3.59	3.72	3.72	3.85	3.98	4.04	4.01	3.97	
	NF	19.62	19.45	19.37	19.33	19.20	19.06	18.95	19.00	
Singapore	PF	0.00	0.00	0.00	0.00	0.00	0.00	0.00	0.00	
	NF	0.01	0.01	0.01	0.01	0.01	0.01	0.01	0.01	
Philippines	PF	7.01	6.84	6.94	7.05	7.19	7.18	7.26	7.31	
	NF	12.85	12.76	12.62	12.56	12.51	12.47	12.36	12.29	
Laos	PF	2.01	2.13	2.12	2.06	2.30	2.33	2.25	2.20	
	NF	16.24	16.05	15.77	15.54	15.33	15.06	14.69	14.46	
Indonesia	PF	35.61	35.10	35.43	36.26	37.46	37.60	37.74	38.45	
	NF	115.20	114.74	113.89	113.49	113.04	112.43	111.30	110.77	
Cambodia	PF	0.84	0.77	0.80	0.86	0.88	0.84	0.87	0.89	
	NF	6.45	6.19	6.01	5.81	5.57	5.44	5.37	5.37	
Brunei	PF	0.04	0.04	0.04	0.04	0.04	0.04	0.04	0.04	
	NF	0.47	0.47	0.47	0.47	0.47	0.47	0.47	0.46

### Independent third-party validation

We utilized an external ground-truth dataset from Xu *et al*.^3^ to avoid circular validation. As the reference samples were originally collected circa 2015, we used the CCDC algorithm to detect and filter out any points that underwent sudden land-cover changes or disturbances between 2015 and 2017. This yielded a refined, independent validation set of 2,685 points (1,711 PF, 870 NF, 90 Others). Assessment of our 2017 map against this set resulted in an Overall Accuracy of 89.65% and a Kappa of 0.79, with the producer’s and user’s accuracies reaching 93.28% and 92.58% for PF, and 82.87% and 94.99% for NF, respectively. This confirms our pipeline reliably discriminates physical forest types while ruling out model overfitting.

### Usage Notes

**Fig. 6:**
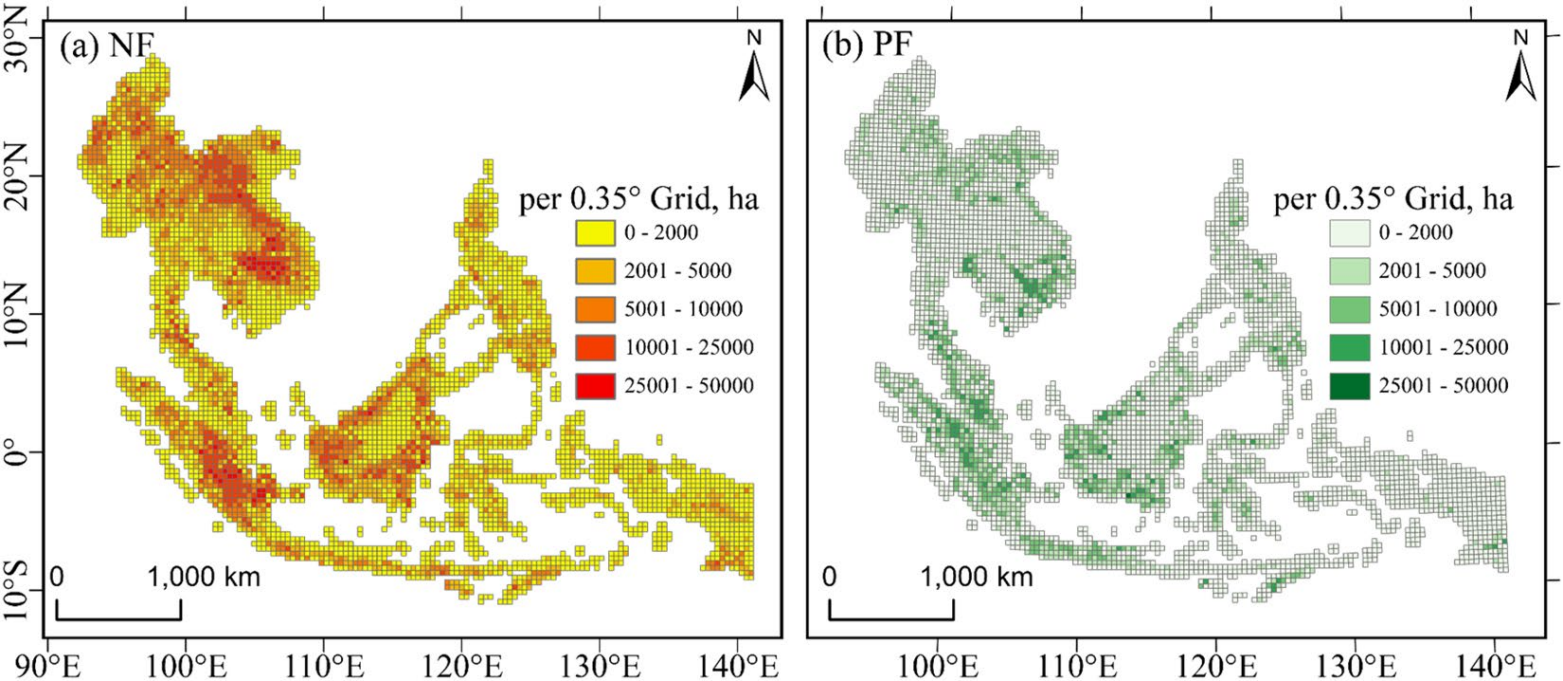
Spatial distribution of loss and gain areas for plantation forests (PF) and natural forests (NF) in SEA. (**a**) NF loss during 2017–2024 in SEA. (**b**) PF gain during 2017–2024 in SEA.

(1) Scaling: Although provided at 10-m resolution, the dataset can support regional-scale forest dynamic assessments via grid aggregation. Figure 6 illustrates pixel-level changes aggregated into a ${0.35}^{\circ }$ grid. (2) Boundary Effects: Users should note that as 2017 and 2024 are the start and end years of the HMM sequence, these years exhibit more substantial boundary effects compared to intermediate years^34,42^. (3) Discrepancy with FAO Definitions: While the dataset captures the relative scale and distribution trends of plantations across countries consistent with FAO statistics (r=0.69), the absolute area estimates for PF are systematically higher than FAO figures (points lie significantly above the 1:1 line). This deviation stems from fundamental differences in definition^43^. The FAO definition of “forest” strictly excludes tree crops grown for agriculture, such as oil palm, rubber, and coconut^41,44^. For instance, Malaysia reports over 5.7 million hectares of oil palm, which are not counted as forest area; similarly, Indonesia classifies vast plantations as non-forest land use. However, from an earth observation perspective, these high-canopy-closure, contiguous oil palm and rubber plantations possess spectral features and physical structures typical of forests^14^. As this dataset aims to provide objective land-cover information, it classifies these widespread economic forests as PF. Users should be aware that this difference in statistical scope (“Land Use” vs. “Land Cover”) leads to the observed numerical deviation.

## Data Availability

The Southeast Asia Plantation and Natural Forests (SEA-PNF) dataset (2017–2024) generated in this study is freely available at the figshare repository under the access address: 10.6084/m9.figshare.30847220).

## Code availability

The source code for sample refinement, Random Forest classification, and Hidden Markov Model (HMM) post-processing is available in the GitHub repository at https://github.com/ruiduobao/plantforest_classify.
